# Sport Psychology: Technologies Ahead

**DOI:** 10.3389/fspor.2020.00010

**Published:** 2020-02-13

**Authors:** Camille Jeunet, Denis Hauw, Jose del R. Millán

**Affiliations:** ^1^CLLE Lab, CNRS, Univ. Toulouse Jean Jaurès, Toulouse, France; ^2^École Polytechnique Fédérale de Lausanne, Geneva, Switzerland; ^3^Institut des Sciences du Sport, Université de Lausanne, Lausanne, Switzerland; ^4^Department of Electrical and Computer Engineering, The University of Texas at Austin, Austin, TX, United States; ^5^Department of Neurology, The University of Texas at Austin, Austin, TX, United States

**Keywords:** training, motor-imagery, brain-computer interface (BCI), brain stimulation, neurophenomenology, virtual reality, sport

## 1. Virtual Reality and Motor Imagery

Several factors may make it difficult, if not impossible, for athletes to train in real sport conditions. These include on the one hand extrinsic factors such as unfavorable weather conditions or costs related to the facilities and materials used, and on the other hand factors intrinsic to the athlete, such as injuries.

When confronted to such situations, it becomes necessary for the athletes to be provided with alternative training solutions in order to maintain their level of performance. In this section, we will introduce two of these alternative solutions, namely virtual reality (VR) and motor imagery (MI). The first alternative training solution we will mention here is Virtual Reality (VR). VR is a computer-based simulation that is both interactive and immersive. It provides the opportunity to design fully controlled training procedures that are both adaptable to each athlete and ecological. Coupled with the fact that VR enables athletes to train in environments that may not be readily accessible, these properties make VR a very relevant and promising training tool to improve sport performance. Several studies have already revealed that athletes who had trained in a virtual environment obtained performances similar to those who had trained in real sport conditions (Bideau et al., 2004, 2010; Vignais et al., 2015) (for a review, see Neumann et al., 2018) suggesting that VR can provide a sufficiently high level of qualitative feedback to be used as a training tool (e.g., see Miles et al., 2012 for a review of virtual environments designed for ball sport training). Nonetheless, Neumann et al. (2018) stress the fact that the benefits of VR for enhancing physiological, psychological and motor performance can be modulated by different factors related to the athletes themselves, but also to the VR system and environment.

The second alternative training solution is Motor Imagery. MI has been defined as a mental representation of an action without any concomitant body movement (Guillot and Collet, 2008). More specifically, Morris et al. (2005) have stated that in sports, MI “may be considered as the creation or recreation of an experience generated from memorial information, involving quasi-sensorial, quasi-perceptual, and quasi-affective characteristics, that is under the volitional control of the imager, and which may occur in the absence of the real stimulus antecedents normally associated with the actual experience.” MI is extensively practiced by athletes, not only when they cannot access real sport conditions, but also as a complementary training procedure or for preparation in competition. Formal studies have repeatedly shown that, indeed, this practice enabled the improvement motor performance (Guillot and Collet, 2008), and notably of strength (Lebon et al., 2010). In the last decade, extensive research has been led in order to better understand (Moran et al., 2019) and model (Guillot and Collet, 2008) the mechanisms underlying the influence that MI has on motor performance, but also to better understand how the sport that is practiced influences MI abilities (Di Corrado et al., 2019), in order then to design efficient training procedures. Nonetheless, the main limitation of current MI training procedures is the absence of feedback. In other words, when they practice MI, athletes are provided with no information regarding their performance—which may be measured, for instance, by the extent to which they modulate their sensorimotor rhythms (SMR). As a consequence, they cannot assess the efficiency of their MI training nor know how to adapt in order to improve this capacity. One promising solution to overcome this limitation consists in measuring the athletes' brain activity while they perform MI, and in providing them with real-time feedback indicating how well they modulate the target brain rhythms, e.g., their SMR. A basic form of such paradigm is called neurofeedback. Next section briefly describes the benefits of technologies called Brain-Computer Interfaces (BCIs), that are based on neurofeedback, and how to modulate brain activity by applying small electrical currents.

## 2. Brain-Computer Interfaces and Brain Stimulation

In a brain-computer interfaces (BCIs), neural signals recorded from the brain are fed into a decoding algorithm that translates these signals into outputs (e.g., Millán, 2019 and references therein). Compared to neurofeedback—where users modulate a single, usually predefined, physiological component—a BCIs rely on multivariate brain feature processing algorithms for feature selection and decoding, which makes it possible to control in real time a variety of brain-actuated devices. Although BCIs are being extensively explored as new control and communication modalities for people with physical disabilities, including new forms of sports (Perdikis et al., 2018), as well as neurorehabilitiation (Biasiucci et al., 2018; Cervera et al., 2018), their use in the framework of sports is still at its infancy. Here we shortly discuss two initial attempts. Jeunet et al. (2018) hypothesized that a BCI based on EEG signatures of athletes' cognitive abilities could help them to train those abilities and contribute to further improve their athletic performance. They present preliminary results on the possibility of using such a kind of BCI aiming to increase soccer goalkeepers' performance through the improvement of their covert visuo-spatial attention (CVSA) abilities—which is key for these athletes and is characterized by modulations of patterns of alpha synchronization over parieto-occipital areas. Our second example combines a BCI with another technology covered in this paper, namely VR. Pereira et al. (2018) investigated EEG correlates of realistic training and competition scenarios while novice shooters were immersed in a 10 m Olympic pistol shooting environment. In the training scenario, shooters remained alone; while in the competition scenario there were other competitors and an audience. Authors found a differential between-subject effect of competition on mu (8–12 Hz) oscillatory activity during aiming; compared to training, the more the subject was able to desynchronize their mu rhythm during competition, the better was their shooting performance. These results provide evidence that mu desynchronization has a positive effect on performance during competition.

Rather than training subjects to learn to modulate their brain rhythms and acquire BCI skills, an alternative is to use brain stimulation techniques, in particular non-invasive modalities such as transcranial direct current stimulation (tDCS). tDCS applies low-amplitude constant currents via scalp electrodes and modify neuronal transmembrane polarization if applied sufficiently long (10–20 min), thereby inducing cortical excitability changes in humans (Nitsche and Paulus, 2000). Modulatory effects of tDCS depend on the current direction: anodal stimulation has an excitatory effect, while cathodal stimulation yields an inhibitory effect. Thus, anodal tDCS over the motor cortex has shown to improve motor learning in healthy subjects (Reis et al., 2009). These initial findings are triggering substantial attention in sports, although early attempts report a mixture of positive (yet without proper control studies) and negative outcomes. As an example, Angius et al. (2016) found that anodal tDCS over the motor cortex led to a reduced perception of effort and increased endurance in amateur cyclist when the cathode was placed on the controlateral shoulder.

## 3. Back to the Beginning: Neurophenomenology

One future challenge is to link EEG with the understanding of various levels of organization of human activity during sports in ecological settings. This means that (a) phenomenological dimensions of athletes' activity (i.e., being ready to move, feeling comfortable when acting, having good sensations when performing) might be taking into account, (b) portable electroencephalogram systems could record athletes' brain signals during performance, and (c) advanced bioengineering techniques to decode these signals in real time as well as analyzing the experiences with advanced phenomenological techniques should be developed. While neurophenomenological methods have already been used to investigate the neural correlates of several sense-making processes in subjective experience in human activities such as meditation or epileptic seizure (e.g., Petitmengin et al., 2006), rare attempts have been made in sports.

In an exploratory study, we combined brain activity using EEG (third person data) with first-person qualitative analysis of pre-reflective experience emerging during a standing split task in dance. We expected that brain activity signatures underlying the consciousness of various efficient ways of being-and-acting in this task would be identified. Data was collected through (a) video tapes of the athletes' behavior while performing the sporting task, (b) video-recorded and transcribed verbalizations and commentaries elicited post-action during self-confrontation interviews, and (c) 64-electrode EEG recordings made during sport performance. Qualitative analyses were performed on the data from the self-confrontation interviews using the course-of-action method, which consists in identifying elementary units of meaning and their components (involvement, focus and general feelings) (e.g., Hauw and Durand, 2007; Hauw, 2018). This method identified different sequences of organization of the athletes' activities. Results illustrated that each participant typically organized their activity around three or more sequences, but that these sequences were very distinct according to their expertise level. Advanced EEG analysis allowed us to study neural activity despite the signal contamination due to movement-related artifacts during the task execution. Preliminary results showed the possibility of extracting neural activity that was correlated with movement preparation (Bereitschafts potential).

In conclusion, despite the emergence of some research on the topic, the field of neurotechnologies for sport psychology is still in its infancy and has to face a number of technological difficulties. The conceptual and methodological design might be a relevant direction for future development for sports learning and training.

## Author Contributions

CJ, DH, and JM all took part in the writing of this opinion paper.

### Conflict of Interest

The authors declare that the research was conducted in the absence of any commercial or financial relationships that could be construed as a potential conflict of interest.
